# Mendelian Randomization Research Examining the Effects of Insomnia on Herpes Zoster and Postherpetic Neuralgia

**DOI:** 10.1155/prm/7106878

**Published:** 2026-08-28

**Authors:** Xueying Yang, Xuan Li, Jingrun Lin, Yuqing Chen, Xuerong Zhang, Qiong Zhao

**Affiliations:** ^1^ Department of Anesthesiology, Sun Yat-Sen Memorial Hospital, Sun Yat-Sen University, Guangzhou 510120, China, sysu.edu.cn

**Keywords:** herpes zoster, insomnia, Mendelian randomization study, postherpetic neuralgia, sleep duration

## Abstract

**Background:**

Insomnia is closely linked to herpes zoster (HZ) and postherpetic neuralgia (PHN); nonetheless, the causal relationship has not been sufficiently clarified. This research employs the Mendelian randomization (MR) method to investigate the causal relationship between insomnia and HZ, as well as PHN.

**Methods:**

Summary statistics from publicly available GWASs were utilized for four sleep traits (insomnia, sleep duration, habitual short sleep [< 7 h/day], and habitual long sleep [≥ 9 h/day]), HZ, PHN, and common risk factors (smoking, alcohol use, obesity, hypertension, and diabetes [Type 1 and Type 2]). Bidirectional univariable two‐sample MR was conducted to assess causal associations between insomnia and HZ/PHN. Multivariable MR (MVMR) was performed to determine whether insomnia exerted an independent causal effect on HZ after adjusting for these risk factors.

**Results:**

This study identified a positive causal effect of insomnia on HZ risk via inverse variance weighted (IVW) (OR = 1.97, 95% confidence interval [CI]: 1.13–3.45, *p* = 0.017, *P*_−FDR_ = 0.034). Longer sleep duration showed a suggestive inverse association with HZ, with each additional hour associated with 34% lower odds (IVW: OR = 0.66, 95% CI: 0.43–1.00, *p* = 0.048; *P*_−FDR_ = 0.096). Neither habitual short sleep (IVW: OR = 1.17, *p* = 0.206) nor habitual long sleep (IVW: OR = 1.11, *p* = 0.446) was causally associated with HZ. No causal relationships were observed between any sleep trait and PHN, nor evidence for reverse causation. In MVMR, insomnia remained associated with HZ after adjusting for diabetes via IVW (OR = 2.36, 95% CI: 1.12–4.93, *p* = 0.023), but this association disappeared after further adjustment for smoking, alcohol use, obesity, and hypertension.

**Conclusion:**

Genetically determined insomnia was causally associated with increased HZ risk, independent of diabetes but probably confounded by smoking, alcohol use, obesity, and hypertension, while longer sleep duration showed a suggestive protective effect.

## 1. Introduction

Insomnia is a common sleep disorder characterized by difficulties initiating or sustaining sleep or experiencing early morning awakening, which negatively impacts quality of life and overall well‐being [1]. Currently, effective treatment methods are lacking [1]. Approximately 30% of adults worldwide report insomnia‐related issues [2]. Emerging evidence indicates that chronic sleep disorders are correlated with impaired immune regulation, heightened susceptibility to infectious diseases, and unfavorable infection outcomes [3–5]. The probability of developing zoster is 1.23 times greater in individuals with sleep disturbances compared to a control group [6].

Herpes zoster (HZ) is an acute infectious dermatological disorder caused by the recurrence of varicella‐zoster virus (VZV). This virus exhibits neurotropic characteristics; following initial infection, it can lead to chickenpox, and upon recovery, it remains inactive within the dorsal root ganglia of the spinal cord or cranial nerve ganglia [7]. Upon a decrease in immune system efficacy, the virus reacts, propagates through nerve fibers to the dermis, and manifests symptoms. The global annual number of HZ cases is approximately 3–5 per thousand, with a notably elevated rate in the Asia‐Pacific region (3–10 cases/1000 individuals) [8]. The prevalence of this disease is elevated in individuals aged over 50 years, and these individuals are more prone to complications such as postherpetic neuralgia (PHN) [7]. This neuralgia is characterized by prolonged duration, intense pain, and compromised sleep quality and may result in challenges with sleep onset or frequent awakenings, contributing to insomnia [7]. A prospective multicenter trial of 261 individuals older than 50 years with HZ monitored for 180 days revealed that acute HZ adversely impacted all aspects of health, especially sleep, which affected 64% of the participants [9].

The associations between insomnia and HZ and PHN have garnered significant attention in recent years. A case–control study established that sleep disturbances significantly increased the likelihood of HZ [10]. A recent study in South Korea indicated that inadequate sleep and stress increase susceptibility to HZ [11]. A higher incidence of HZ is observed when insomnia is combined with depression [5]. A lack of sleep is associated with PHN progression but not with HZ risk, according to community‐based prospective cohort research [12]. Observational studies may be confounded by factors such as age, sex, comorbidities, medication use, immune function, lifestyle, socioeconomic status, and education level, which can complicate the ability to establish definitive causal inferences. Randomized controlled trials (RCTs) represent the optimal methodology for establishing causal relationships; however, research on rare diseases often necessitates larger sample sizes and is characterized by significant time demands, labor intensity, and difficulties in securing ethical approval. From this particular interpretive perspective, Mendelian randomization (MR) studies provide evidence that may support the use of genetic variants as instrumental variables (IVs) to assess potential causal relationships. Since alleles are inherited in what appears to be a largely irreversible process, this method substantially reduces confounding factors and tends to avoid issues of reverse causality. What appears to emerge from this evidence is that it utilizes existing genome‐wide association studies (GWASs), is cost‐effective, has a large sample size, and has seemingly been widely used in causal inference studies [13, 14].

## 2. Methods

### 2.1. Study Design

**FIGURE 1:**
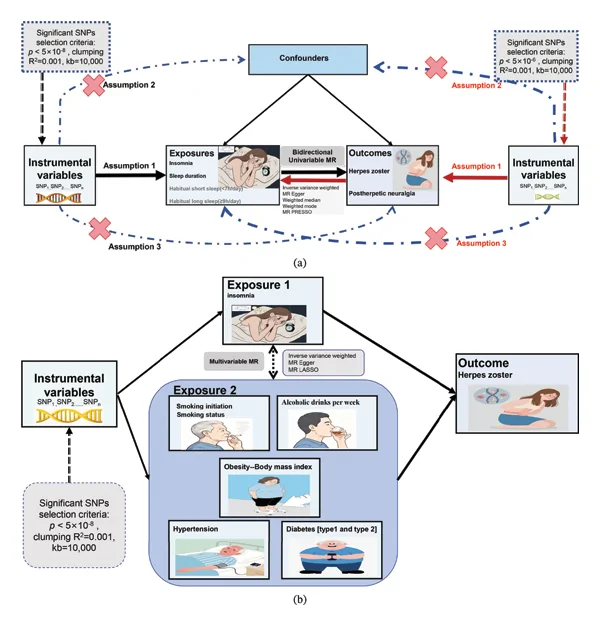
Basic design of the MR study. (a) The univariable bidirectional MR, thick black arrows indicating forward MR and thick red arrows indicating reverse MR. Three key assumptions underpin the validity of genetic variants as instrumental variables: (1) a robust association with the exposure (relevance); (2) no association with confounders (independence); and (3) an exclusive pathway from genetic variant to outcome mediated only by the exposure of interest (exclusion restriction or absence of pleiotropy). (b) The multivariable MR, which examines whether insomnia retained an independent causal effect on HZ after adjusting for common risk factors, including smoking (smoking initiation and smoking status), alcohol use (alcohol drinks per week), obesity (body mass index), hypertension, and diabetes (Type 1 and Type 2), separately. Abbreviations: MR, Mendelian randomization; MR‐PRESSO, MR pleiotropy residual sum and outlier; MR‐LASSO, Mendelian randomization least absolute shrinkage and selection operator; SNP, single‐nucleotide polymorphism.

In line with the STROBE‐MR guidelines (Table S1) [15], the analytical design of our study is outlined in the flowchart presented in Figure 1. In forward MR, four sleep traits (insomnia, sleep duration, habitual short sleep [< 7 h/day], and habitual long sleep [≥ 9 h/day]) were exposures, with HZ and PHN as outcomes (Figure 1a). Reverse MR was performed with HZ/PHN as exposures and insomnia/sleep duration as outcomes; habitual short/long sleep was excluded due to incomplete GWAS data (Figure 1a). MVMR assessed whether the causal effect of insomnia on HZ was influenced by common risk factors (smoking, alcohol use, body mass index [BMI], hypertension, and diabetes [Type 1 and Type 2]) (Figure 1b).

**FIGURE 2:**
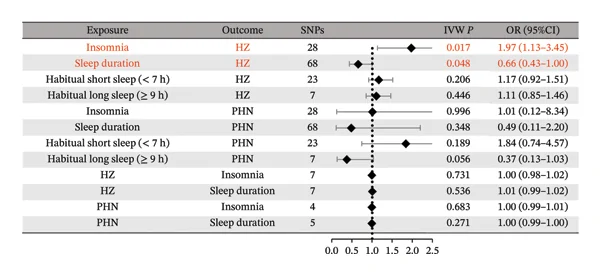
Forest plot of the IVW results of the univariable bidirectional MR study. Abbreviations: MR, Mendelian randomization; IVW, inverse variance weighted; HZ, herpes zoster; PHN, postherpetic neuralgia; SNPs, single‐nucleotide polymorphisms; OR, odds ratio; CI, confidence interval.

**FIGURE 3:**
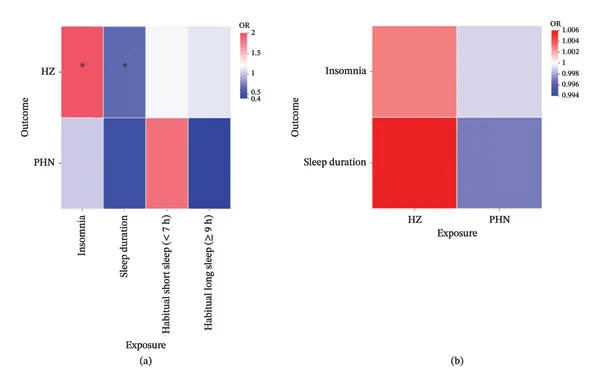
Heatmap of IVW assessments of univariable bidirectional MR study. ^∗^ *p* < 0.05. Abbreviations: MR, Mendelian randomization; IVW, inverse variance weighted; HZ, herpes zoster; PHN, postherpetic neuralgia.

We subsequently screened IVs associated with exposure, ensuring that all IVs met the criteria of three assumptions: (1) IVs must demonstrate a strong association with the exposure; (2) IVs must not link to any confounders that could influence both the exposure and outcome; and (3) in addition to their relevance to exposures, IVs must not impact the outcome directly [14]. In this study, the selection of single‐nucleotide polymorphisms (SNPs) as IVs was subject to the following criteria: a *p*‐value of less than 5 × 10^−8^ for the four sleep‐related traits and common risk factors serving as exposures; and a *p*‐value of less than 5 × 10^−6^ for HZ and PHN serving as exposures, given that no SNPs were identified when the *p*‐value was set at less than 5 × 10^−8^ or 5 × 10^−7^. Additionally, the linkage disequilibrium (LD) threshold was set at 10,000 kb, and the *R* ^2^ value was required to be less than 0.001. In addition, we calculated the F value (*F* = *β* ^2^/se^2^) for validation purposes to evaluate their correlation to verify that the IVs accurately represented the exposure components [16, 17]. An intense association was considered to exist when the F value was greater than 10. After harmonizing effect alleles throughout the GWAS for exposure and outcome, two‐sample and univariable MR tests, as well as multivariable Mendelian randomization (MVMR) analyses, were conducted, along with sensitivity analyses. R software (Version 4.3.3) as well as R packages “TwoSampleMR,” “MR PRESSO,” and “MR Radial” were used to perform all the statistical calculations. To address potential bias arising from multiple comparisons, a false discovery rate (FDR) correction method was applied. A possible connection was assessed when the *P*_−FDR_ was greater than 0.05 and *p* was less than 0.05 (see Figures 2 and 3).

### 2.2. Data Resources

Four representative sleep problems were selected for a comprehensive investigation of sleep‐related issues: insomnia, sleep duration, habitual short sleep (defined as < 7 h/day), and habitual long sleep (defined as ≥ 9 h/day). Summary statistics regarding insomnia and sleep duration were sourced from the U.K. Biobank (UKB, https://www.nealelab.is/uk-biobank). Summary statistics for insomnia were obtained from a GWAS of 336,965 European‐ancestry individuals, comprising 10,894,596 SNPs. Insomnia was assessed via the automated computer‐assisted examination (ACE) touchscreen questionnaire (Data‐Field 113241). Participants were asked: “Do you have trouble falling asleep at night or do you wake up in the middle of the night?” with instructions to refer to the past 4 weeks if sleep patterns varied. Responses were recorded on a 4‐point ordinal scale: “Never/rarely,” “Sometimes,” “Usually,” and “Prefer not to answer.” Genetic association data related to sleep duration included 460,099 European‐ancestry individuals and 9,851,867 SNPs from UKB. Sleep duration was self‐reported at baseline using the ACE touchscreen questionnaire (Data‐Field 1160): “About how many hours sleep do you get in every 24 h? (please include naps)”; participants reported the average over the past 4 weeks if patterns varied. Real‐time quality control rejected values < 1 or > 23 h and flagged values < 3 or > 12 h for confirmation. Responses coded as “Do not know” (−1), “Prefer not to answer” (−3), or otherwise implausible were excluded. Furthermore, we utilized specific sleep duration grouping data to strengthen the validation. Habitual short sleep (< 7 h/day, *n* = 106,192) and habitual long sleep (≥ 9 h/day, *n* = 34,184) were analyzed using GWAS summary data from Dashti et al., derived from the same UKB assessment but with sleep duration dichotomized against a common reference group of moderate sleepers (7–8 h/day, *n* = 446,118) [18]. Both datasets utilized the same ACE touchscreen questionnaire (Data‐Field 1160) for sleep duration assessment at baseline. Moreover, these self‐reported definitions were supported by accelerometer‐derived sleep estimates. The study identified 27 and 8 genome‐wide significant loci for short and long sleep at a rigorous criterion for screening (*p* < 5 × 10^−8^), respectively.

For HZ, the FinnGen consortium recently updated the summary genetic statistics (Release 12; https://r12.finngen.fi/), encompassing information from a total of 487,448 European individuals, which included 7132 cases and 480,316 healthy participants. The endpoint was defined using the Finnish Hospital Discharge Register and Cause of Death Register. Cases were identified by ICD‐10 code B02 (Zoster [herpes zoster]), ICD‐9 code 053, or ICD‐8 code 053, encompassing all HZ diagnoses and subtypes [19]. For PHN, genetic association data were retrieved from the same FinnGen release (Release 12; https://r12.finngen.fi/), which encompasses a total of 435,861 individuals of European descent, comprising 490 patients and 435,371 healthy volunteers. The endpoint was defined as postzoster neuralgia using the Finnish Hospital Discharge Register. Cases were identified by ICD‐10 code G53.0 (postzoster neuralgia) or B02.2 (zoster with other nervous system involvement, including B02.21–B02.29) [19].

In addressing potential confounders, factors such as smoking, alcohol use, obesity, hypertension, and diabetes appear to represent the most common risk factors for both insomnia and HZ [20–25]. Within this broader analytical framework, GWAS summary data for smoking initiation (involving 311,629 cases and 321,173 controls) and alcoholic drinks per week (from 335,394 samples) were sourced from the GSCAN consortium [26]. Concurrently, data pertinent to smoking status were collected from a cohort of 468,170 Europeans [27]. Genetic instruments for obesity, specifically BMI, were derived from a substantial sample of 681,275 European individuals through the GIANT consortium [28]. Summary statistics for hypertension (comprising 129,909 cases and 354,689 controls) [29], Type 1 diabetes (with 18,942 cases and 501,638 controls) [30], and Type 2 diabetes (including 62,892 cases and 596,424 controls) [31] were obtained from several meta‐GWAS studies.

The genetic data utilized in this investigation were sourced from publicly available databases. The study protocol was approved by the Institutional Review Board (IRB) of Sun Yat‐sen Memorial Hospital (approval number: SYSEC2‐2025‐BA‐895). To minimize population bias, the datasets chosen were exclusively from European populations. The features of the selected dataset are presented in detail in Table 1.

**TABLE 1:** Details of the GWASs included in the Mendelian randomization.

Phenotype	Consortium or PMID	Year	Participants (case/control [n])	Number of SNPs	GWAS ID
Insomnia	Neale Lab	2017	336,965	10,894,596	ukb‐a‐13
Sleep duration	MRC‐IEU	2018	460,099	9,851,867	ukb‐b‐4424
Habitual short sleep (< 7 h/day)	30846698	2019	411,934 (106,192/305,742)	14,661,600	NA
Habitual long sleep (≥ 9 h/day)	30846698	2019	339,926 (34,184/305,742)	14,661,600	NA
Herpes zoster	Finn Consortium	2024	487,448 (7132/480,316)	16,380,433	finngen_R12_AB1_ZOSTER
Postherpetic neuralgia	Finn Consortium	2024	435,861 (490/435,371)	16380406	finngen_R12_G6_POSTZOST
Smoking initiation	GSCAN/30643251	2019	607,291 (311,629/321,173)	11802365	ieu‐b‐4877
Smoking status	29892013	2018	468,170	11,973,425	ebi‐a‐GCST90029014
Alcoholic drinks per week	GSCAN/30643251	2019	335,394	11,887,865	ieu‐b‐73
Body mass index	GIANT/30124842	2018	681,275	2,336,260	ieu‐b‐40
Hypertension	33959723	2021	484,598 (129,909/354,689)	9,587,836	ebi‐a‐GCST90038604
Type 1 diabetes	34012112	2021	520,580 (18,942/501,638)	59,999,551	ebi‐a‐GCST90014023

*Note:* GSCAN, GWAS and Sequencing Consortium of Alcohol and Nicotine use; GIANT, Genetic Investigation of ANthropometric Traits consortium.

Abbreviations: MRC‐IEU, Medical Research Council Integrative Epidemiology Unit; NA, not applicable.

### 2.3. Univariable MR Analysis

To explore potential MR estimates of exposures and outcomes, our investigation employed a set of five distinct MR approaches, namely, the inverse variance weighted (IVW), MR‐Egger, weighted median, weighted mode, and MR‐PRESSO methods. Multiple approaches have been employed, as they tend to rely on different underlying assumptions regarding horizontal pleiotropy. The primary analytical method employed was IVW meta‐analysis of the Wald ratio for individual SNPs, which assumes that the instruments influence the outcome exclusively through the exposure of interest and not via any other route [32]. Moreover, the MR‐Egger [33] and weighted median [34] methods were employed as supplementary analyses. These approaches are potentially more robust across a wider range of situations, although they generally have lower statistical efficiency, resulting in wider confidence intervals (CIs). The weighted mode method calculates the effect value of every SNP on exposure and outcome, assigns weights to them, and subsequently identifies the most prevalent weighted effect value as the apparent value of the causal effect. More theoretically, this method is robust against outliers and horizontal pleiotropy but may be less precise than IVW and potentially more precise than MR‐Egger [34]. MR‐PRESSO is generally employed to explore and correct the outliers that seem to be caused by horizontal pleiotropy [35]. What follows from this analysis is that after identifying such outliers, they are typically excluded or corrected, and the causal effect is re‐estimated to obtain more robust results. However, in light of these methodological considerations, when the number of IVs is small or the effect estimates between IVs vary substantially, overcorrection may occur, which appears to lead to biased estimates of the causal effect [35].

### 2.4. Sensitivity Analysis

Sensitivity analysis plays a crucial role in the TSMR, enabling the detection of potential pleiotropy and heterogeneity. A suite of tests, including Cochran’s *Q* test [36], the MR‐Egger regression test [33], and MR‐PRESSO [37], was applied. When MR sensitivity analysis shows no pleiotropy and no heterogeneity, the MR uses a fixed‐effects model for estimation to provide the most reliable results. If there is no pleiotropy and possible confounding SNPs have been removed, but heterogeneity still exists, a random‐effects model analysis is needed, and the MR results are less reliable. If pleiotropy exists, outliers must be further removed, and MR statistics recalculated; otherwise, the results will be unreliable. The leave‐one‐out method enables us to intuitively assess whether the MR statistical results are significantly influenced by the effects of one or more SNPs, thereby affecting the reliability of the results.

### 2.5. MVMR Analysis

To assess the independent causal effect of insomnia on HZ, MVMR was conducted adjusting for common risk factors (smoking, alcohol use, obesity, hypertension, and diabetes [Type 1 and Type 2]) [37]. Three MVMR methods were employed: MR‐IVW, MR‐Egger, and Mendelian Randomization least absolute shrinkage and selection operator (MR‐LASSO). Heterogeneity was evaluated using the IVW Q‐test and MR‐Egger intercept test.

## 3. Results

### 3.1. Screening of Genetic Instruments

In the forward MR analyses, 28 independent SNPs were identified as IVs for insomnia, whereas 68, 23, and 7 SNPs were linked to sleep duration, habitual short sleep (< 7 h/day), and habitual long sleep (≥ 9 h/day) at a rigorous criterion for screening (*p* < 5 × 10^−8^), respectively (Table S2), with HZ and PHN as outcomes. The F‐statistic values for individual SNPs, as presented in Table S2, ranged from 16 to 224, with mean values of 39, 43, 27, and 30 for the exposures mentioned above, respectively. These selected SNPs were considered valid and strong genetic instruments because their F values all exceeded the threshold of 10.

In the reverse MR analyses, HZ and PHN were analyzed as exposures and insomnia and sleep duration as outcomes; no IVs could be identified under stringent significance thresholds (*p* < 5 × 10^−8^ or *p* < 5 × 10^−7^). Therefore, a relaxed threshold of *p* < 5 × 10^−6^ was applied, yielding 7 (HZ ⟶ insomnia, F‐statistic range: 21–32), 7 (HZ ⟶ sleep duration, F‐statistic range: 21–32), 4 (PHN ⟶ insomnia, F‐statistic range: 21–24), and 5 (PHN ⟶ sleep duration, *F*‐statistic range: 21–24) valid IVs, respectively (Table S2). All F‐statistics exceeded 10, indicating that the selected SNPs possessed adequate statistical strength even with the *p* < 5 × 10^−6^ threshold, and the risk of weak‐instrument bias was low. All IVs ultimately included in the analysis passed the directionality test (Steiger *p* value < 0.05), ruling out reverse causality (Table S2).

For MVMR, insomnia and each risk factor were included as co‐exposures, with HZ as the outcome. Instrument selection followed stringent criteria (kb = 10,000; *R* ^2^ = 0.001; *p* < 5 × 10^−8^), yielding 141, 53, 466, 218, and 145 SNPs for models adjusting for smoking (initiation and status), alcohol consumption, BMI, hypertension, and diabetes (Types 1 and 2), respectively. All corresponding F‐statistics exceeded 10.

### 3.2. Causal Effect of Insomnia on HZ

Analysis of the 28 insomnia‐related SNPs revealed that severe insomnia was positively correlated with the incidence of HZ (IVW: OR = 1.97, 95% CI = 1.23–3.45, *p* = 0.017, *P*_−FDR_ = 0.034; MR‐PRESSO: OR = 1.97, 95% CI = 1.23–3.15, *p* = 0.009, *P*_−FDR_ = 0.018). The results are consistent with those obtained from the MR‐Egger (OR = 1.21, 95% CI = 0.25–5.93, *p* = 0.820), weighted median (OR = 1.45, 95% CI = 0.65–3.26, *p* = 0.364), and weighted mode methods (OR = 1.23, 95% CI = 0.39–3.87, *p* = 0.731) (Table S3). Subsequent MR‐Egger analyses and Cochran’s *Q* tests revealed no substantial pleiotropy or heterogeneity (*p* > 0.05). The leave‐one‐out method confirmed that no SNP significantly drove the MR results (Table S4, Figure S1).

The MR‐Egger method allows for directional pleiotropy but has lower statistical power than IVW. In this study, although the Egger result was nonsignificant (*p* = 0.820), its intercept test was also nonsignificant (*p* = 0.524), suggesting that this inconsistency stemmed from insufficient power rather than pleiotropy, which was further corroborated by MR‐PRESSO (*p* = 0.871). Additionally, while the weighted median did not reach statistical significance (*p* = 0.364), its effect direction was consistent with IVW and MR‐PRESSO, supporting the assumption that the majority of IVs were valid, ruling out weak instrument bias, and enhancing confidence in the reliability of the IVW estimate.

### 3.3. Causal Effect of Sleep Duration on the HZ

Genetically predicted sleep duration was potentially inversely associated with HZ risk, with each 1‐h/day increase corresponding to a 34% reduction in HZ odds (IVW: OR = 0.66, 95% CI = 0.43–1.00, *p* = 0.048, *P*_−FDR_ = 0.096). Consistent results were obtained via the MR‐Egger method (OR = 0.37, 95% CI = 0.07–1.92, *p* = 0.239), the weighted median approach (OR = 0.64, 95% CI = 0.34–1.19, *p* = 0.156), and MR‐PRESSO (OR = 0.78, 95% CI = 0.28–2.17, *p* = 0.632) (Table S3), although none of the associations reached statistical significance. Additionally, sensitivity analysis revealed the absence of heterogeneity (Cochran *Q* test: *p* = 0.222) and pleiotropy (MR‐PRESSO: *p* = 0.257) (Table S4, Figure S2).

### 3.4. Causal Effects From Habitual Short Sleep (< 7 h/day) and Habitual Long Sleep (≥ 9 h/day) to HZ

The IVW analysis revealed no causal relationship between habitual short sleep (< 7 h/day) (OR = 1.17, 95% CI = 0.92–1.51, *p* = 0.206) or habitual long sleep (≥ 9 h/day) (OR = 1.11, 95% CI = 0.85–1.46, *p* = 0.446) and HZ (Table S3). These results are consistent with findings from the MR‐Egger, weighted median, weighted mode, and MR‐PRESSO methods. Sensitivity analysis indicated no evidence of heterogeneity or pleiotropy (Table S4, Figures S3‐S4). Specifically, a shorter sleep duration (< 7 h/day) seems to increase the risk of HZ, whereas a longer sleep duration (≥ 9 h/day) does not mitigate this risk.

### 3.5. Causal Effects of Four Sleep Traits on PHN

The IVW method revealed no clear causal evidence of the four genetically predicted sleep traits on PHN: insomnia (OR = 1.01, 95% CI: 0.12–8.34, *p* = 0.996), sleep duration (OR = 0.49, 95% CI: 0.11–2.20, *p* = 0.348), habitual short sleep (< 7 h/day) (OR = 1.84, 95% CI: 0.74–4.57, *p* = 0.189), or habitual long sleep (≥ 9 h/day) (OR = 0.37, 95% CI: 0.13–1.03, *p* = 0.056) (Table S3). Although the MR‐PRESSO results suggested the presence of pleiotropy for habitual long sleep (≥ 9 h/day) on PHN (*p* = 0.040), no outliers were detected using the MR‐Radial approach (Figure S5). Furthermore, the evidence was insufficient to indicate obvious heterogeneity regarding the link between the four sleep traits and PHN (Table S4 and Figures S6–S9).

### 3.6. Causal Effect of HZ, PHN, on Insomnia and Sleep Duration

The IVW analyses indicated the absence of a meaningful causal relationship between genetically proxied HZ (OR = 1.00, *p* = 0.731) or PHN (OR = 1.01, *p* = 0.683) and insomnia (Table S3, Figures S10–S11). In parallel, HZ (OR = 1.01, *p* = 0.536) and PHN (OR = 1.00, *p* = 0.271) likewise, exerted no discernible influence on sleep duration (Table S3, Figures S12–S13). Sensitivity analyses (including MR‐Egger regression, weighted median, and MR‐PRESSO) were consistent with the IVW results and verified the stability of the results (Table S3). Finally, there is no persuasive evidence of substantial heterogeneity and pleiotropy across HZ and PHN when examined in relation to insomnia and sleep duration (Table S4).

### 3.7. MVMR Assessing the Independent Causal Effect of Insomnia on HZ After Adjusting for Risk Factors

MVMR was employed to mutually analyze the effects of insomnia and common risk factors on the risk of HZ. After adjusting for diabetes (including Type 1 and Type 2 diabetes), insomnia is directly associated with an increased risk of HZ (IVW: OR 2.36, 95% CI: 1.12–4.93, *p* = 0.023). This finding was consistent with the estimates from MR‐Egger (OR 2.36, 95% CI: 1.13–4.95, *p* = 0.023) and MR‐LASSO (OR 2.36, 95% CI: 1.12–4.93, *p* = 0.023) (Table 2), and no significant heterogeneity (Cochran’s *Q* test: IVW, *p* = 0.539; MR‐Egger, *p* = 0.533) or pleiotropy (Egger intercept = −0.002, *p* = 0.383) was detected (Table S4). However, when smoking (IVW: OR 1.74, 95% CI: 0.96–3.12, *p* = 0.066), alcohol intake (IVW: OR 1.60, 95% CI: 0.88–2.92, *p* = 0.126), obesity (IVW: OR 0.79, 95% CI: 0.45–1.37, *p* = 0.396), or hypertension (IVW: OR 1.62, 95% CI: 0.88–2.98; *p* = 0.118) was adjusted for separately, the above association disappeared (Table 2). Pleiotropy and heterogeneity analyses further confirmed the robustness of these results (Table S4).

**TABLE 2:** Multivariable Mendelian randomization estimates of the associations from insomnia on herpes zoster.

Method	N. SNPs	*p* value	OR (95% CI)
Multivariable MR adjusting for smoking initiation and smoking status			
Multivariable IVW	141	0.066	1.74 (0.96, 3.12)
Multivariable MR‐Egger	141	0.134	1.59 (0.87, 2.90)
Multivariable MR‐LASSO	141	0.066	1.74 (0.96, 3.12)
Multivariable MR adjusting for alcoholic drinks per week			
Multivariable IVW	53	0.126	1.60 (0.88, 2.92)
Multivariable MR‐Egger	53	0.157	1.56 (0.84, 2.87)
Multivariable MR‐LASSO	53	0.126	1.60 (0.88, 2.92)
Multivariable MR adjusting for body mass index			
Multivariable IVW	466	0.396	0.79 (0.45, 1.37)
Multivariable MR‐Egger	466	0.454	0.81 (0.46, 1.42)
Multivariable MR‐LASSO	466	0.396	0.79 (0.45, 1.37)
Multivariable MR adjusting for hypertension			
Multivariable IVW	218	0.118	1.62 (0.88, 2.98)
Multivariable MR‐Egger	218	0.135	1.59 (0.87, 2.93)
Multivariable MR‐LASSO	218	0.118	1.62 (0.88, 2.98)
Multivariable MR adjusting for diabetes [Type 1 and Type 2]			
Multivariable IVW	145	0.023	2.36 (1.12, 4.93)
Multivariable MR‐Egger	145	0.023	2.36 (1.13, 4.95)
Multivariable MR‐LASSO	145	0.023	2.36 (1.12, 4.93)

*Note:* N. SNPs, number of single‐nucleotide polymorphisms used in MR.

Abbreviations: CI, confidence interval; IVW, inverse variance weighted; MR, Mendelian randomization; OR, odds ratio.

## 4. Discussion

This MR study demonstrated that genetically predicted insomnia conferred susceptibility to HZ—an association independent of diabetes yet substantially attenuated after adjustment for tobacco use, alcohol intake, excess body weight, and hypertension—whereas prolonged sleep duration exhibited nominally protective effects. However, no clear evidence of a causal effect was detected between insomnia and PHN. Additionally, reverse causation between HZ or PHN and either insomnia or sleep duration was not supported by the evidence.

The association between insomnia and HZ has received increased scrutiny in recent years. A case–control study involving individuals aged 65–66 years (389 cases and 511 matched controls) revealed an obvious association between HZ and sleep disorders, with an adjusted OR of 2.52 [10]. A 3‐year cohort study involving a Taiwanese population, which included 131,001 participants comprising patients with sleep disorders without sleep apnea and matched controls, revealed that the incidence of HZ was 1.23 times greater in patients with sleep disorders than in controls (95% CI: 1.17–1.30) [6]. The mean prevalence of HZ among insomniacs was 2.77 cases per 1000 person‐years, which was significantly greater than the prevalence in the control group of 1.81 cases per 1000 person‐years (AHR = 1.62, 95% CI = 1.35–1.93), according to a 13‐year retrospective cohort study carried out in Taiwan (47,256 healthy controls vs. 31,504 age‐ and sex‐matched insomniacs) [5]. Individuals with a history of insomnia spanning fewer than 4 years (AHR: 6.69, 95% CI: 4.44–9.39), 4–6 years (AHR: 4.42, 95% CI: 3.07–6.36), or more than 6 years (AHR: 1.38, 95% CI: 1.14–1.87) presented significantly elevated risks of HZ [5]. Furthermore, depression significantly elevated the risk of HZ when associated with insomnia, presenting a 4.95‐fold increase compared with the normal control group [5]. The results align with these observations. A prospective cohort study involving 12,329 Japanese community residents, with a follow‐up period of up to 3 years, found that insufficient sleep was associated with the development of PHN (HR = 2.02, 95% CI: 1.06–3.85) but not with the risk of HZ onset [12]. The inconsistencies in these observational studies indicate the need for additional validation research. MR studies conducted in U.K. and Finnish populations revealed that insomnia significantly increased susceptibility to influenza (OR_-IVW_ = 1.65, *p* = 5.86 × 10^−7^), upper respiratory tract infections (URIs) (OR_-IVW_ = 1.94, *p* = 8.14 × 10^−31^), and COVID‐19 infection (OR_-IVW_ = 1.08, *p* = 0.037), as well as the risk of COVID‐19 hospitalization (OR_-IVW_ = 1.47, *p* = 4.96 × 10^−5^) [38]. These findings underscore the critical role of adequate sleep in sustaining immune function, with chronic sleep deprivation identified as a significant contributor to viral infections. Prior observational studies have faced challenges in addressing the impact of unmeasured confounding factors, including educational attainment, comorbid conditions, environmental influences, and lifestyle choices. In contrast, our findings evaluate causal relationships rather than solely correlational associations, demonstrating that insomnia symptoms may be a significant risk factor for zoster.

The physiological mechanisms linking insomnia to HZ remain incompletely understood. Sleep serves as a crucial regulator of immune responses, and sleep deprivation compromises immune function, increasing vulnerability to infections [39, 40]. Insomnia leads to alterations in circulating immune cell defensive capacity, including diminished natural killer cell migration and activity [38], increased proinflammatory monocyte activity [41], elevated CRP, IL‐6, and TNF‐α levels, and NF‐κB inflammatory pathway activation [41]. Additionally, peripheral blood neutrophil counts increase with concomitant inflammatory factor release and decreased phagocytic activity [42]. T‐cell activity is reduced, with weakened proliferative capacity and impaired antigen presentation [42]. Furthermore, peripheral blood B cells migrate to the brain, resulting in increased brain B‐cell abundance [43]. Experimental sleep deprivation (4 h per day for 4 consecutive days) significantly diminishes immune responses to influenza vaccination, particularly IgG antibody concentrations [44]. Insomnia severity is positively correlated with morning cortisol levels (*r* = 0.37, *p* = 0.03) [45], and elevated cortisol suppresses immune cell activity, increasing infection vulnerability. Insomnia is also linked to the onset and progression of chronic illnesses, including cardiovascular disease [46], depression [47], diabetes [47], and cancer [48], which can impair immune function and increase HZ susceptibility [49, 50].

This study systematically assessed horizontal pleiotropy through multiple complementary methods. In univariable MR, MR‐Egger intercept, MR‐PRESSO, and Cochran’s *Q* tests found no significant pleiotropy or heterogeneity (all *p* > 0.05, see Table S4), indicating that IVW estimates were not biased by directional pleiotropy. In MVMR, Egger intercepts across adjusted models were likewise nonsignificant (*p*: 0.189–0.614), further excluding pleiotropic interference. Notably, the causal effect of insomnia on HZ persisted after adjusting for diabetes (OR = 2.36, *p* = 0.023) but disappeared after adjusting for smoking, alcohol, obesity, or hypertension individually (IVW *p* > 0.05). This differential pattern was unlikely driven by pleiotropy, as all MVMR models showed nonsignificant pleiotropy tests. Hence, it suggests that genetically predicted insomnia may increase HZ risk through pathways that substantially overlap with lifestyle‐related immunometabolic factors, rather than through a causal pathway entirely independent of these comorbidities. On the one hand, diabetes may impair VZV‐specific cell‐mediated immunity through metabolic‐immune cross‐talk, including advanced glycation end product‐induced inflammation and insulin resistance‐related T‐cell dysfunction [51]. This pathway appears partially distinct from insomnia‐related neuroendocrine dysregulation, as the insomnia‐HZ association remained robust after diabetes adjustment. On the other hand, smoking, alcohol use, obesity, and hypertension may act as either confounders or mediators (or both) in the insomnia‐HZ relationship. Chronic smoking depletes naive T‐cell pools and impairs IFN‐γ production [52]; alcohol disrupts intestinal barrier integrity and induces innate immune exhaustion [53]; obesity promotes chronic low‐grade inflammation and T‐cell dysfunction [54]; and hypertension shares sympathetic nervous system overactivation with insomnia [55]. These factors exhibit potential genetic and phenotypic correlations with insomnia, and their individual adjustment in MVMR eliminated the insomnia effect. This observation is consistent with recent MR evidence suggesting that adiposity traits (BMI, body fat percentage) demonstrate robust causal effects on HZ [56], whereas sleep traits alone showed null associations—an inconsistency potentially explained by the older FinnGen release (Release 10) used in that study, which may have yielded lower statistical power than our current analysis based on Release 12. Crucially, the absence of MVMR in that study precludes disentanglement of whether the null insomnia effect represents genuine lack of causality or residual confounding by genetically correlated BMI‐related traits.

This study has several limitations. Initially, all GWAS data were obtained from Europeans. Further investigation is needed to determine whether our findings are consistent across other populations. Additionally, while we considered common confounding factors, unmeasured confounders may have impacted the observed associations. Third, the extensive administration of the zoster vaccine has further decreased the already low incidence of HZ and PHN. The incidence of cases within the European population is limited, with 7132 cases of HZ and 490 cases of PHN reported among 430,000 participants. Our conclusions would benefit from validation through larger sample sizes. Asian populations have a broader demographic base and a somewhat higher incidence of HZ; however, comprehensive GWASs are lacking. Further investigation is advised to fill this gap.

## 5. Conclusion

Genetically predicted insomnia was associated with increased HZ risk, an effect that persisted independent of diabetes but was substantially attenuated after adjustment for smoking, alcohol use, obesity, and hypertension. Longer sleep duration demonstrated nominally protective effects. These findings suggest that insomnia‐related HZ susceptibility may operate through immunometabolic pathways shared with lifestyle comorbidities, highlighting the need for integrated prevention strategies targeting both sleep optimization and lifestyle improvement.

## Author Contributions

The article was drafted, revised, and critically reviewed by Xueying Yang, Xuerong Zhang, Xuan Li, Jingrun Lin, Yuqing Chen, and Qiong Zhao.

## Funding

No funding was received for this manuscript.

## Disclosure

All authors affirm that this research was conducted independently, with no apparent business or monetary affiliations that could be perceived as potential conflicts of interest. They decided on the journal to which the manuscript was submitted and gave their final permission for the version to be published. They also take responsibility for every facet of the work.

## Conflicts of Interest

The authors declare no conflicts of interest.

## Supporting Information

Additional supporting information can be found online in the Supporting Information section.

## Supporting information


**Supporting Information** Supporting information for this study includes the STROBE‐MR reporting standards checklists, a summary table of all MR analysis results, a detailed table of IVs, and a table of heterogeneity and pleiotropy tests. Additionally, for each exposure‐outcome pair, we provide scatter plots, forest plots, funnel plots, leave‐one‐out plots, and MR‐Radial outlier detection plots, totaling 13 supporting figures.

## Data Availability

The raw data supporting the findings of this study can be found in the references cited [19–21, 28–33], which are sourced from publicly available GWAS summary data resources (https://gwas.mrcieu.ac.uk/, http://www.nealelab.is/uk-biobank, https://www.finngen.fi/fi/hyodynna_tuloksia). The secondary analysis data supporting the findings of this study can be found in the Supporting Information of this manuscript.
